# Phenotyping Refractory Cardiogenic Shock Patients Receiving Venous–Arterial Extracorporeal Membrane Oxygenation Using Machine Learning Algorithms

**DOI:** 10.31083/j.rcm2508303

**Published:** 2024-08-22

**Authors:** Shuo Wang, Liangshan Wang, Zhongtao Du, Feng Yang, Xing Hao, Xiaomeng Wang, Chengcheng Shao, Jin Li, Hong Wang, Chenglong Li, Xiaotong Hou

**Affiliations:** ^1^Center for Cardiac Intensive Care, Beijing Anzhen Hospital Capital Medical University, 100029 Beijing, China

**Keywords:** cardiogenic shock, venous–arterial extracorporeal membrane oxygenation, machine learning, phenotype

## Abstract

**Background:**

This study used machine learning to categorize cardiogenic shock (CS) patients treated with venous–arterial extracorporeal membrane oxygenation (VA-ECMO) into distinct phenotypes. Subsequently, it aimed to clarify the wide mortality variance observed in refractory CS, attributing it to the condition’s inherent heterogeneity.

**Methods:**

This study enrolled a cohort of CS patients who received VA-ECMO support. By employing rigorous machine learning (ML) techniques, we generated and validated clusters based on determinants identified through algorithmic analysis. These clusters, characterized by distinct clinical outcomes, facilitated the examination of clinical and laboratory profiles to enhance the understanding of patient responses to VA-ECMO treatment.

**Results:**

In a study of 210 CS patients undergoing VA-ECMO treatment, 70.5% were male with a median age of 62, ranging from 53 to 67 years. Survival rates were 67.6% during VA-ECMO and 49.5% post-discharge. Patients were classified into three phenotypes based on the clinical and laboratory findings: “platelet preserved (I)”, those with stable platelet counts, “hyperinflammatory (II)”, those indicating significant inflammation, and “hepatic–renal (III)”, those showing compromised liver and kidney functions. Mortality rates (25.0%, 52.8%, and 55.9% for phenotypes I, Ⅱ, and Ⅲ, respectively (*p* = 0.005)) varied significantly among these groups, highlighting the importance of phenotype identification in patient management.

**Conclusions:**

This study identified three distinct phenotypes among refractory CS patients treated using VA-ECMO, each with unique clinical characteristics and mortality risks. Thus, highlighting the importance of early detection and targeted intervention, these findings suggest that proactive management could improve outcomes for those showing critical signs.

## 1. Introduction 

The mortality rate of cardiogenic shock (CS) is as high as 50% [1]. Venous–arterial extracorporeal membrane oxygenation (VA-ECMO), though not yet validated by randomized clinical trials for efficacy, has been used increasingly for refractory CS, with survival outcomes reported between 16% and 42% [2, 3, 4]. This highlights the role of VA-ECMO as a critical, albeit temporary, support mechanism in managing severe CS cases.

CS is a heterogeneous clinical condition followed by chronic heart failure or an acute cardiac injury, such as acute myocardial infarction (AMI), myocarditis, malignant ventricular arrhythmia, cardiac arrest, or even pulmonary dysfunctions. The complexity of the etiology and clinical profiles accompanied by extracorporeal membrane oxygenation (ECMO) also leads to heterogeneity [5]. Clinical outcomes have been proven to be linked to lactate behavior, platelet count, organ function, and inflammation [6, 7, 8, 9, 10]. These heterogeneities make clinical practice more difficult and limit our ability to develop new strategies in “nonspecific” populations.

Several scoring systems for VA-ECMO, including the survival after VA-ECMO score (SAVE), prEdictioN of Cardiogenic shock OUtcome foR Acute myocardial infarction patients salvaGed by VA-ECMO score (ENCOURAGE), and pRedicting mortality in patients undergoing veno–arterial Extracorporeal MEMBrane oxygenation after coronary artEry bypass gRafting (REMEMBER) scores, have been developed. These tools aim to predict outcomes and identify patients most likely to benefit from VA-ECMO by analyzing pre-ECMO parameters. These systems enhance prognosis prediction and decision-making for patients facing cardiogenic shock after acute myocardial infarction or undergoing coronary artery bypass grafting, utilizing the availability of specific clinical indicators before ECMO initiation [3, 10, 11]. However, previous attempts have yet to characterize patients receiving VA-ECMO adequately. Thus, a deeper exploration and understanding of the disease’s heterogeneity, beyond the causes of CS and initial ECMO parameters, could enable clinicians to identify distinct patient phenotypes. This, in turn, may facilitate the development of novel therapeutic strategies.

Machine learning (ML) methodologies have been applied to delineate complex clinical conditions such as acute respiratory distress syndrome (ARDS), sepsis, and CS by segmenting data into distinct datasets [12, 13, 14]. This research employs ML to investigate the heterogeneity among CS patients treated using VA-ECMO, analyzing their clinical, biological, and inflammatory profiles to categorize them into unique sub-phenotypes. Such a nuanced approach aims to deepen our understanding of CS physiology under VA-ECMO management, pinpoint specific patient subgroups for targeted clinical interventions, and potentially evolve into a sophisticated risk assessment tool for clinical use.

## 2. Materials and Methods

### 2.1 Patient Population

This study was a single-center, observational study approved by the institutional ethics review board (IRB) at Beijing Anzhen Hospital (202102X). International Research Database for Extracorporeal Support (approval date: February 23, 2021; study title: International Research Database for Extracorporeal Support). All procedures were conducted in alignment with the ethical guidelines of the overseeing ethics committee on human experimentation and conformed to the 1975 Helsinki Declaration. Before collecting clinical samples (e.g., blood), informed consent was secured to analyze the demographic, physiological, and hospital outcome data. Participants, or, when applicable, their relatives, were briefed on the anonymity of data collection and provided the option to opt out of the study.

The study enrolled adult CS patients who received VA-ECMO for circulatory support. CS is defined as follows [15]: (1) systolic blood pressure <90 mmHg for 30 min, mean arterial pressure <65 mmHg for 30 min, or the need for vasopressors to achieve a blood pressure of 90 mmHg; (2) pulmonary congestion or elevated left ventricular filling pressure; (3) signs of impaired organ perfusion with at least one of the following indications: altered mental status, cold, clammy skin, oliguria, or increased serum lactate despite optimized supportive measures, such as an intra-aortic balloon pump and inotropes. Moreover, patients presenting with cardiogenic shock after initial cardiac surgery were also included; however, patients diagnosed with pulmonary embolism requiring ECMO were eliminated.

### 2.2 VA-ECMO Management

Details regarding VA-ECMO initiation and management have been described previously [16]. The ECMO team members performed all VA-ECMO procedures. Successful ECMO weaning was defined as the lack of obvious hemodynamic deterioration for at least 48 h after removing VA-ECMO support (more details are provided in Appendix 1).

### 2.3 Selection of Cluster-Determined Variables

Baseline characteristics were recorded within the initial 24 hours after intensive care unit (ICU) admission, including age, sex, body mass index (BMI), laboratory test after 24 hours of VA-ECMO initiation (including complete blood count, hepatic-renal, and coagulation function), and arterial blood gas (the worst value before VA-ECMO initiation, 4 hours, and 24 hours after VA-ECMO initiation). The inflammatory response is widely recognized as a key factor influencing patient outcomes in ECMO therapy, as evidenced by previous studies [10, 17, 18, 19, 20]. Accordingly, plasma levels of interleukin-6 (IL-6) and interleukin-10 (IL-10) were quantified using the Luminex multiplex assay (PPX-15, Assay ID: MXMFX3N, Thermo Fisher Scientific, Waltham, MA, USA). The specific time points for these measurements, set at 24 hours post-ECMO initiation, adhered to the established protocol for blood sample collection at our center. Moreover, treatment details such as body temperature during VA-ECMO, VA-ECMO peak flow, pre-ECMO left ventricular ejection fraction (LVEF), left ventricular diastolic diameter, and mean arterial pressure were also recorded. The use of vasoactive agents was described as the vasoactive inotropic score (VIS, Appendix 1).

Given the limited number of cases, a semi-supervised machine-learning algorithm was applied to select cluster-determined variables. Parameters included in the clustering algorithm were associated with clinical outcomes based on previous research [6, 8, 21] or according to clinical experience. Detailed approaches are shown in Appendix 1. The variables with the highest predictive value were chosen as cluster-determined variables and subsequently used to define the optimal number of clusters (k).

### 2.4 Consensus k-Means Algorithm Analysis

Consensus k-means algorithm analysis is a classic ML technique used in previously reported research to identify the homogeneity of a specific disease. Before starting the consensus k-means analysis, the number of clusters (k) should be ascertained (a detailed algorithm is shown in Appendix 1). All the main machine-learning steps were carried out using R-software on RStudio (Version 2021.09.1+372, Posit Software, PBC, Boston, MA, USA).

To assess the clustering efficacy of our dataset, we adopted a quantitative methodology. Our analysis utilized the cluster package in R, aiming to calculate the silhouette width for each observation, which indicates the clustering performance. The process involved the computation of Euclidean distances between each pair of observations using the dist. function. Combined with the clustering outcomes, these distances were analyzed using the silhouette function. The silhouette method offers a graphical summary of the classification accuracy for each object. Specifically, the silhouette width of an observation quantifies its similarity to its cluster (cohesion) versus its dissimilarity to other clusters (separation).

We calculated the average silhouette width by averaging these values across all observations. This average is a measurable gauge of clustering effectiveness, where higher averages indicate more distinctly defined clusters.

### 2.5 Statistics Analyses

Lilliefor’s test was used to analyze normality. Normal variables were described using the mean, non-normal variables by the median, and qualitative variables by proportion. A confidence interval of 95% was used to estimate dispersion measures. Since the quantitative variables possessed more than 2 groups, ANOVA or Kruskal–Wallis analyses were used depending on normality, and post hoc analyses were performed using the Mann–Whitney test with significance correction, respectively. The chi-square test was used for qualitative variables alongside post hoc analysis using Bonferroni correction. A *p* < 0.05 was considered the cut-off point for statistical significance. The superscripts a, b, and c indicate significant pairwise differences among the clusters. Statistical analyses were conducted utilizing RStudio, a front-end interface for R software (R version 4.2.0 (2022-04-22 ucrt)), and validation was carried out using the Statistical Package for the Social Sciences (SPSS) (Version 25.0, IBM, New York, USA). The data were visualized using RStudio on R-software (R version 4.2.0 (2022-04-22 ucrt)) and Prism 8 (Version 8.0.2(263), GraphPad Software, LLC, San Diego, CA, USA).

## 3. Results

### 3.1 Patient Characteristics

Between January 2018 and May 2021, 282 patients receiving ECMO at the Beijing Anzhen Hospital were screened, and 210 patients were eventually recruited and analyzed for this study. Seventy-two patients were excluded for the following reasons: age <18 years (17), acute respiratory failure treated with veno-venous ECMO (4), VA-ECMO duration <24 hours (6), severe missing clinical data (7), and failure to obtain informed consent (6).

**Fig. 1.**
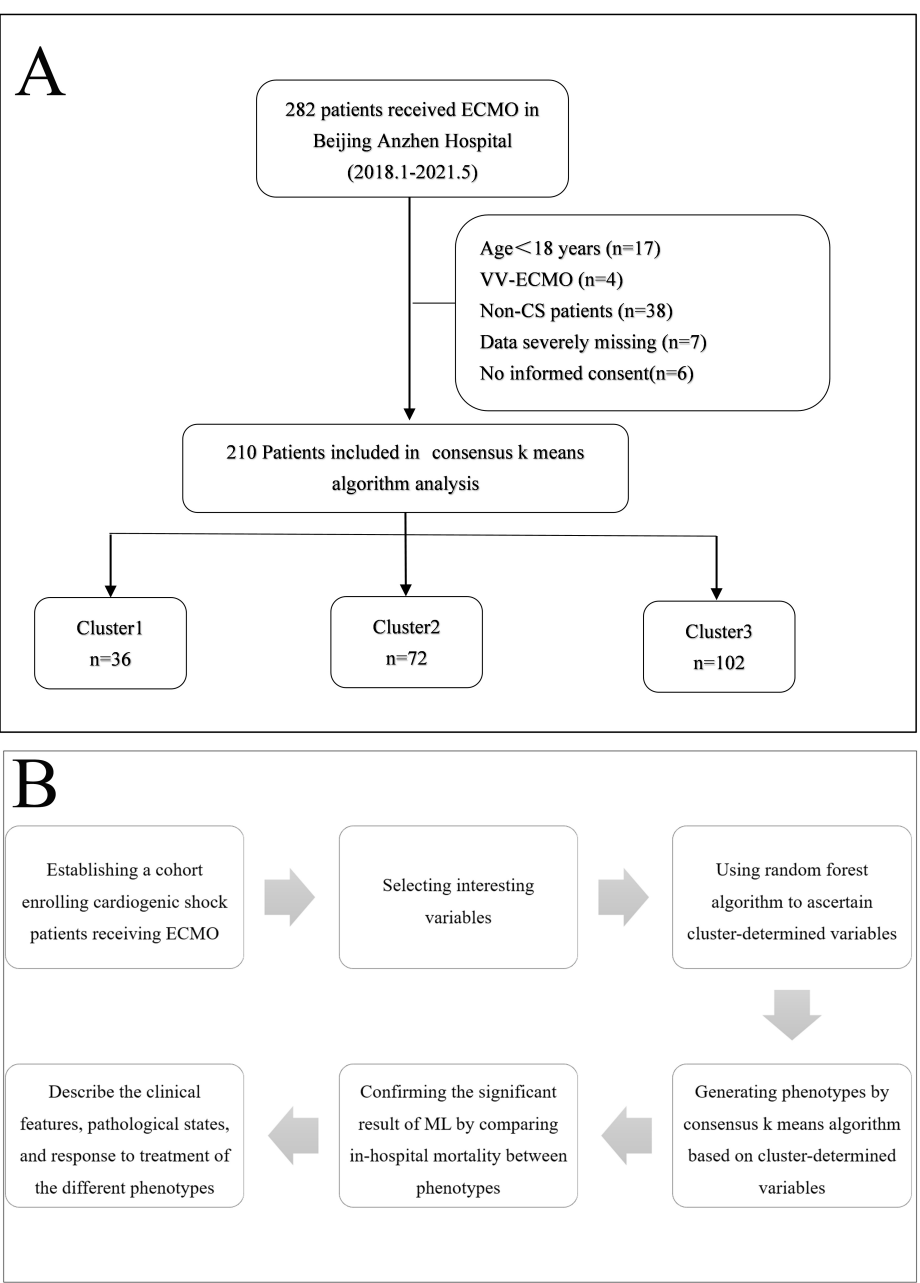
**Flow diagram**. Selection process for participants (A) and machine learning techniques (B) application. Fig. 1A Flow diagram illustrates the process of constructing the patient cohort and the subgroups generated by the machine learning algorithm. Fig. 1B Flow diagram displays the specific logic process and steps of the machine learning algorithm. ECMO, extracorporeal membrane oxygenation; VV-ECMO, veno-venous extracorporeal membrane oxygenation CS, cardiogenic shock; ML, machine learning.

Consequently, the study progressed with a focused cohort of 210 patients diagnosed with CS and treated using VA-ECMO, who were comprehensively analyzed in this investigation as illustrated in Fig. 1A. The median patient age was 62 years (interquartile range (IQR): 53–67 years). The study included 148 (70.5%) men. The rates of successful weaning from VA-ECMO and in-hospital mortality were 67.6% and 49.5%, respectively. The baseline characteristics of the patients are presented in Table 1. The etiology of CS (some patients had multiple diagnoses) included coronary artery disease (109 (51.9%)), valvular heart disease (81 (38.6%)), congenital heart disease (4 (2%)), myocarditis (8 (4%)), and aortic artery dissection (13 (6%)). A total of 144 patients presented with post-cardiotomy CS and received VA-ECMO. The median duration of VA-ECMO was 105.4 h (IQR: 66.7–153.6).

**Table 1.** **Patient characteristics**.

Characteristics		All patients (n = 210)	Phenotype I (n = 36)	Phenotype II (n = 72)	Phenotype III (n = 102)	*p* value
Age, years, median (IQR)		62 (53–67)	60 (52–68)	63 (55–66)	62 (51–68)	0.987
BMI, median (IQR)		24.7 (22.7–27.0)	23.6 (22.2–26.1)	24.5 (22.3–27.8)	24.8 (22.8–26.0)	0.810
Male, n (%)		148 (70.5)	27 (75) ^a^	58 (80.6) ^a^	63 (61.8) ^b^	0.022
Diagnosis, n (%) ^c^						
	Coronary artery disease	109 (51.9)	19 (52.8)	40 (55.6)	50 (49.0)	0.692
	Acute myocardial infarction	20 (9.5)	5 (13.9)	4 (5.6)	11 (10.8)	0.379
	Valvular heart disease	81 (38.6)	17 (47.2)	27 (37.5)	37 (36.3)	0.497
	Congenital heart disease	4 (2)	0 (0)	0 (0)	4 (3.9)	0.104
	Myocarditis	8 (4)	2 (5.6)	2 (2.8)	4 (3.9)	0.795
	Aortic artery dissection	13 (6)	1 (2.8)	7 (9.7)	5 (4.9)	0.348
	Others ^d^	17 (8.1)	3 (8.3)	4 (5.6)	10 (9.8)	0.623
Comorbid conditions, n (%)						
	Hypertension	110 (52.4)	18 (50)	41 (56.9)	51 (50)	0.633
	Diabetes	48 (22.9)	7 (19.4)	17 (23.6)	24 (23.8)	0.607
	Hyperlipidemia	54 (25.7)	5 (13.9)	21 (29.2)	28 (27.5)	0.209
History of myocardial infarction, n (%)		27 (12.9)	6 (16.7)	8 (11.1)	13 (12.7)	0.733
History of cardiac intervention, n (%)		40 (19.0)	5 (13.9)	18 (25)	17 (16.7)	0.258
History of cardiac surgery, n (%)		26 (12.7)	4 (11.1)	12 (16.7)	10 (9.8)	0.429
Cardiac surgery in this hospitalization, n (%)		184 (87.6)	27 (75.0) ^a^	70 (97.2) ^b^	87 (85.3) ^a^	0.003
Surgery under CPB, n (%)		144 (68.6)	22 (61.1)	57 (79.2)	65 (63.7)	0.055
ECPR, n (%)		25 (11.9)	4 (11.1)	7 (9.7)	14 (13.7)	0.727
Combined treatments, n (%)						
	CRRT	104 (49.5)	5 (13.9) ^a^	43 (59.7) ^b^	56 (54.9) ^b^	<0.001
	IABP	132 (62.9)	19 (52.8)	47 (65.3)	66 (64.7)	0.387
Outcomes						
	In-hospital mortality	104 (49.5)	9 (25.0) ^a^	38 (52.8) ^b^	57 (55.9) ^b^	0.005
	Successful weaning from VA-ECMO	142 (67.6)	30 (83.3)	45 (62.5)	67 (65.7)	0.078

a, b: Based on the result of the Bonferroni method after the chi-square test or the result of the LSD method after the nonparametric test, the same letters in the horn markers manifested no significance between phenotypes, while different letters in the horn markers indicated statistically significant. c: Among 109 patients diagnosed with coronary artery disease, 14 combined with valvular disease, and 8 of 13 patients diagnosed with aortic artery dissection combined with coronary artery disease. d: Other diagnoses included cardiogenic shock caused by various arrhythmias, refractory cardiogenic shock after cardiac surgery, and cardiogenic shock caused by pneumonia combined with heart failure. IQR, interquartile range; BMI, body mass index; CPB, cardiopulmonary bypass; ECPR, extracorporeal cardiopulmonary resuscitation; CRRT, continuous renal replacement therapy; IABP, intra-aortic balloon pump; VA-ECMO, venous–arterial extracorporeal membrane oxygenation; LSD, Least Significant Difference.

### 3.2 Clusters Identification

Fig. 1B illustrates the specific logic and procedural steps of the machine learning algorithm. Subsequently, we detail the algorithm’s critical steps.

**Fig. 2.**
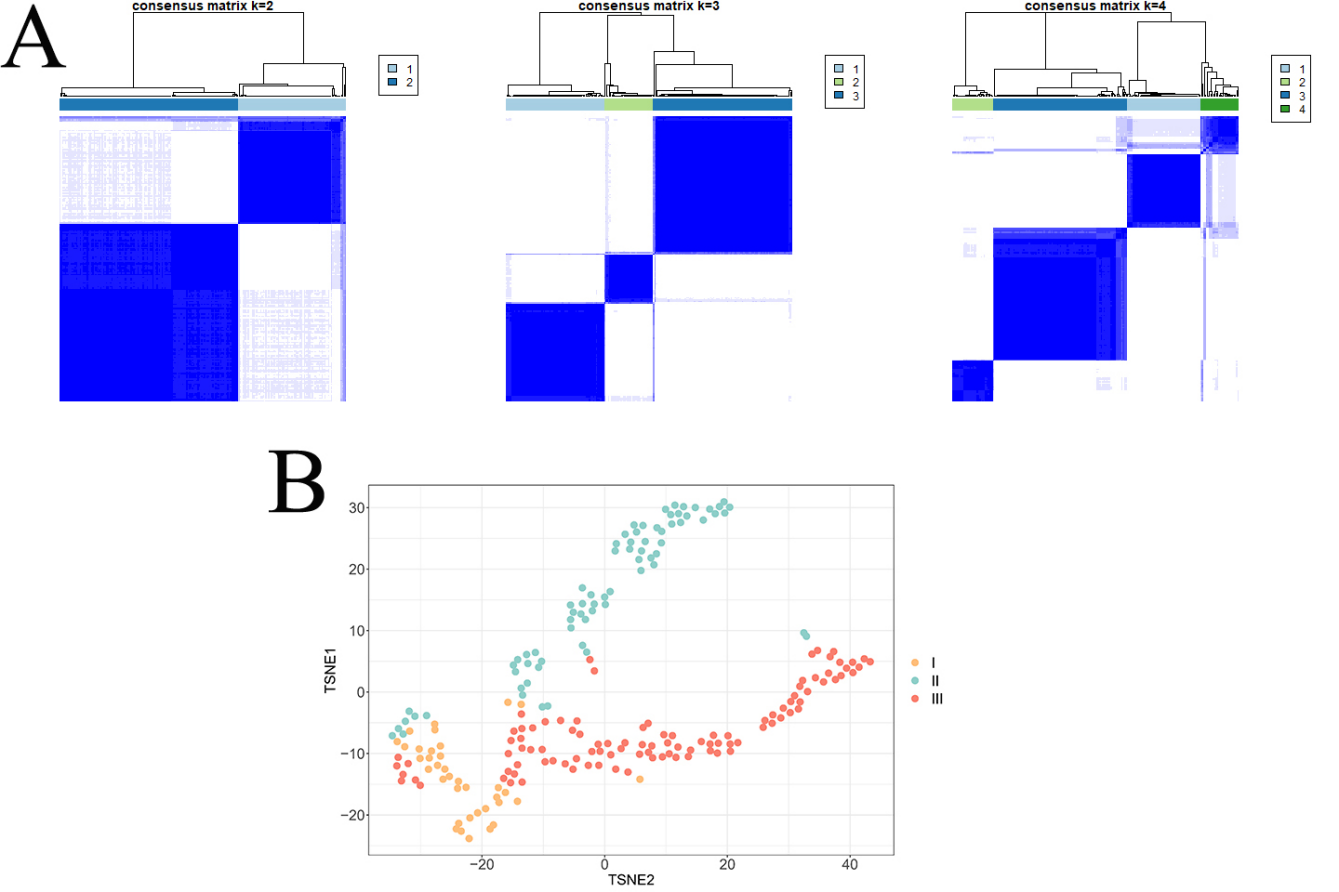
**Selecting the number of clusters**. (A) Comparison of plot graphs with k (number of clusters) = 2, 3, and 4; each column represents one patient, whereas each row displays the assigned clusters. “Sharply marginated” squares indicate stable clusters. K = 3 shows the highest cluster stability. (B) t-distributed stochastic neighbor embedding (TSNE) plot showed a reduction in the dimensionality of the characteristics of the three clusters.

A random forest classifier determined the five highest predictive-value variables (aspartic acid transaminase (AST), 24-hour lactate level, prothrombin time (PT), IL-6, and platelet count), which were included in further cluster analyses (detailed procedures are shown in Appendix 2). Consensus k-means clustering algorithm analysis revealed that k = 3 had the highest cluster stability (Fig. 2A); this result has also been verified by other ML metrics (Appendix Figs. 5,6,7,8). The t-distributed stochastic neighbor embedding (TSNE) plot visualized the distinct differences among the three clusters (Fig. 2B).

The computed average silhouette width of 0.5453 suggests a moderate to high degree of clustering efficacy in the dataset. Values approaching 1 denote well-clustered data points, with clear distinctions between clusters. Conversely, values nearing 0 indicate data points positioned at cluster boundaries, while negative values denote misclassifications.

**Fig. 3.**
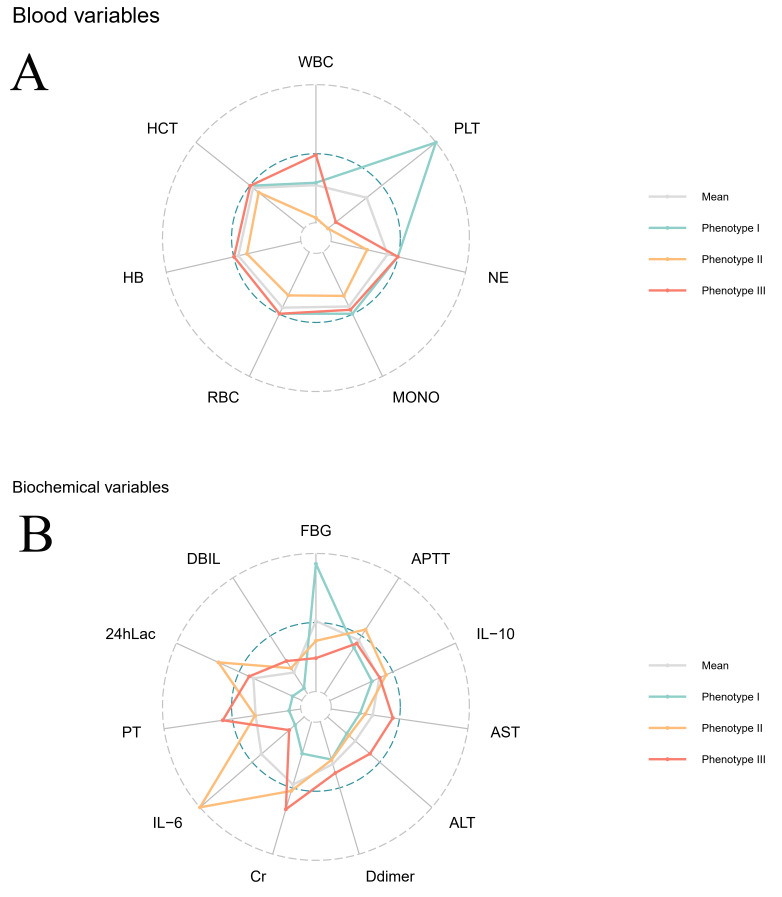
**Radar plots**. Blood routine (A) and biochemical examination (B) of phenotypes. Radar plots illustrate the characters of blood routine and biochemical examination of each cluster. WBC, white blood cell count; PLT, platelet count; NE, neutrophil count; MONO, monocyte count; RBC, red blood cell count; HB, hemoglobin; HCT, hematocrit; FBG, fibrinogen; APTT, activated partial thromboplastin time; IL-10, interleukin-10; AST, aspartic acid transaminase; ALT, alanine aminotransferase; Cr, creatinine; IL-6, interleukin-6; PT, prothrombin time; 24hLac, arterial lactate levels after 24 hours of VA-ECMO initiation; DBIL, direct bilirubin; VA-ECMO, venous–arterial extracorporeal membrane oxygenation.

Consensus k-means clustering algorithm analysis generated three distinct clusters with statistically different in-hospital mortality, suggesting the effectiveness of the clustering algorithm (Table 1). A total of 36 (17.1%), 72 (34.3%), and 102 (48.6%) patients were classified into Phenotype I, II, and III, respectively. Radar plots (Fig. 3) show the deviation of the major laboratory tests (standardized values). According to the clinical profiles, the three most distinctive phenotypes were “platelet preserved (I)”, “hyperinflammatory (II)”, and “hepatic–renal (III)”.

### 3.3 Distinctive Features of Phenotypes

**Fig. 4.**
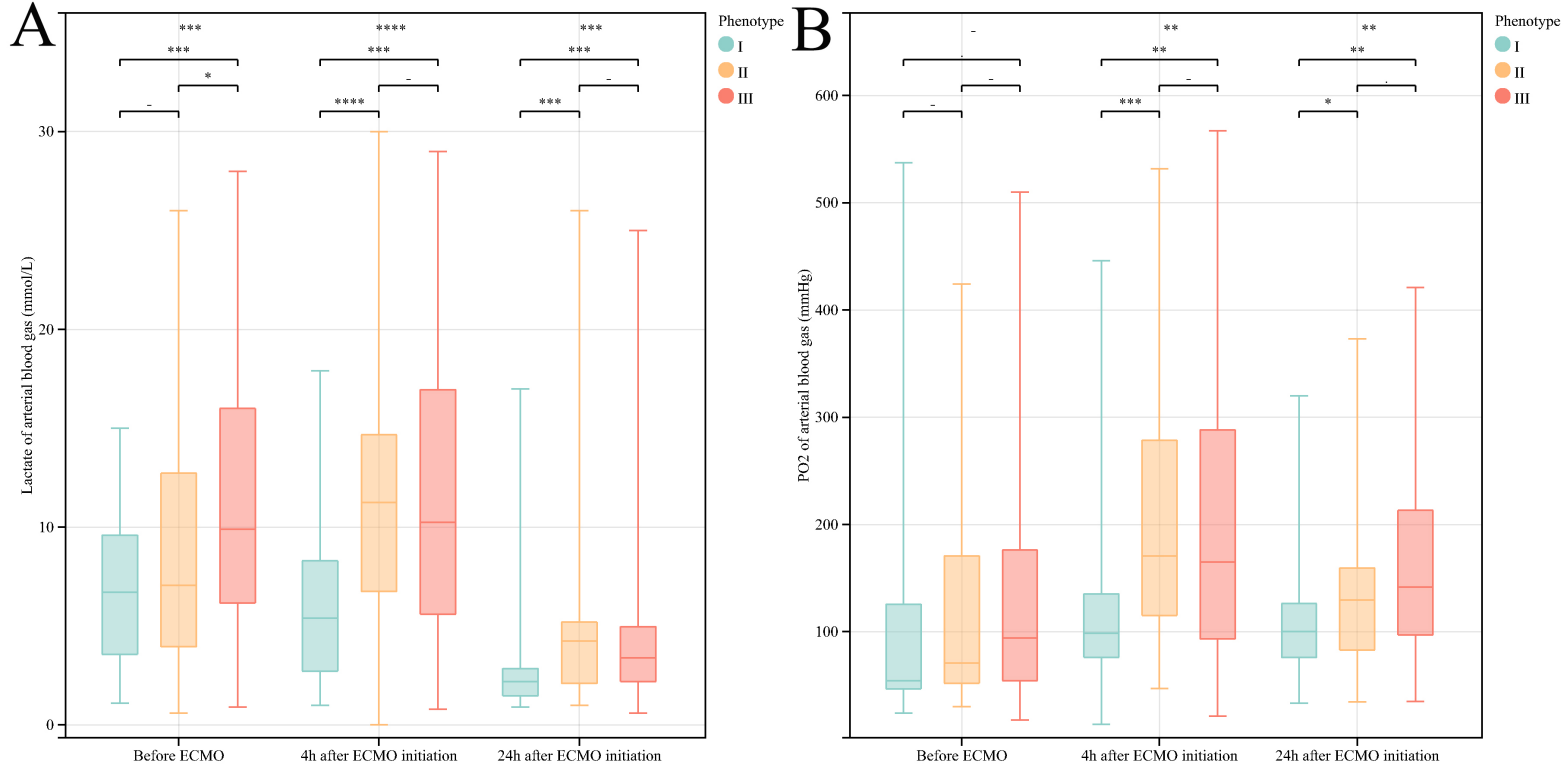
**Phenotype reactions to VA-ECMO**. (A,B) show the dynamic changes in lactate level and PO_2_ among the three phenotypes, respectively, indicating a separate status towards ECMO support. *p < 0.05, **p < 0.01, ***p < 0.001. VA-ECMO, venous–arterial extracorporeal membrane oxygenation; ECMO, extracorporeal membrane oxygenation; PO_2_, partial pressure of oxygen.

The “platelet preserved (I)” phenotype had the most preserved quantity of platelets and the highest fibrinogen (FBG) level, the lowest level of the inflammatory-related indicators (IL-6 and IL-10), and preferable liver and kidney functions after VA-ECMO initiation (Appendix Table 2). Therefore, patients seldom needed continuous renal replacement therapy (CRRT) (13.9%) during VA-ECMO support. Compared with the other two phenotypes, fewer patients underwent cardiac procedures (75.0%), especially coronary artery bypass grafting (22.2%) (Appendix Table 5). This phenotype also had the lowest Sepsis-related Organ Failure Assessment (SOFA) scores (Appendix Table 2) and showed a more sensitive reaction toward VA-ECMO support according to dynamic lactate changes in arterial blood gas examinations (Fig. 4A).

The “hyperinflammatory (II)” phenotype mainly consisted of male patients manifesting a statistically significant increase in inflammatory indicators, such as IL-6 and IL-10 (Appendix Table 3, Fig. 3B). The activated partial thromboplastin time (APTT) of this phenotype was significantly prolonged compared to the others, and the liver function was worse than that of phenotype I but better than phenotype III (Appendix Table 3). In comparison, renal function was in the same poor condition as in phenotype III. There was no significant difference in the reaction towards VA-ECMO support compared to phenotype III (Appendix Table 3, Fig. 4, Appendix Fig. 9).

The “hepatic–renal (III)” phenotype had poor liver function (elevated AST, alanine aminotransferase (ALT), PT, direct bilirubin (DBIL), and FBG levels) (Appendix Table 3) and the highest serum creatinine level among the clusters; it was less responsive towards VA-ECMO support (24 h after VA-ECMO initiation) in terms of eliminating lactate and oxygen consumption (Fig. 4).

The tendencies of the median standardized values of the cluster-determined variables are shown in Appendix Fig. 10.

### 3.4 Outcomes

The in-hospital mortality rates of phenotypes I, II, and III were 25.0%, 52.8%, and 55.9%, respectively. Compared to Phenotype I, patients in Phenotype II had higher mortality (odds ratio (OR), 2.3 [95% confidence interval (CI), 1.2–4.4]), whereas those in Phenotype III (OR, 2.8 [95% CI, 1.4–5.4]) had the highest mortality. There were no significant differences in the other secondary outcomes among phenotypes, such as length of hospital and ICU stay, bleeding complications, and limb ischemia (Appendix Table 4).

## 4. Discussion

In our study, ML algorithms were employed to examine CS patients undergoing VA-ECMO, thereby exploring the disease heterogeneity for the first time. Through rigorous cluster analysis, we delineated three patient groups with unique clinical characteristics, inflammatory responses, and prognoses, termed “platelet preserved (I)”, “hyperinflammatory (II)”, and “hepatic–renal (III)” phenotypes. These insights underscore the diversity among CS patients receiving VA-ECMO and highlight the potential for tailored management strategies based on distinct patient profiles.

In this cohort, the majority of CS patients were men with coronary artery disease, many of whom had undergone cardiac interventions. The in-hospital mortality rate was 49.5%, aligning with previous estimates of 43% to 60% [22, 23, 24, 25, 26]. This study highlights the significant variability in in-hospital mortality among CS patients treated using VA-ECMO, underlining the complexity and heterogeneity of their clinical conditions. Such diversity underscores the limitations of a singular predictive approach, emphasizing the need for multifaceted strategies to enhance prognostic accuracy.

ML, such as k-means algorithm analysis and latent class analysis (LCA), have been implemented to define the distinct clinical phenotypes of diseases. For instance, Calfee *et al*. [27] used clinical and biological data from two ARDS randomized controlled trials and applied LCA to identify two distinct phenotypes; a series of research even proved that these subtypes appeared to have different benefits in distinct fluid and pharmacotherapeutic strategies [12, 28]. These heterogeneities were also widely studied during the coronavirus epidemic [29, 30]. In this study, we applied the consensus k-means clustering algorithm, following rigorous determination and verification of the number of clusters. The achieved average silhouette width of 0.5453 indicates a moderate to high clustering efficacy, suggesting that the clusters are well-defined and cohesive. Most data points were accurately assigned to their respective clusters, demonstrating satisfactory performance in clustering. This foundational analysis is pivotal for subsequent qualitative evaluations and enhancements of the clustering methodology, with the goal of improving data categorization precision and effectiveness in subsequent research.

We analyzed the clinical manifestations, inflammatory profiles, and prognosis of CS patients receiving VA-ECMO support in detail after clustering analysis to generalize the heterogeneous characteristics of distinct phenotypes.

In this cohort, the “platelet preserved (I)” phenotype represented a preserved platelet count, correlating with a favorable prognosis. Thrombocytopenia and platelet dysfunction are common in patients with ECMO, regardless of the ECMO mode. It has been demonstrated that more than 20% have platelet counts lower than 150 × 10^9^/L during VA-ECMO [9]. External circuit surfaces and high shear stress during ECMO are vital in platelet activation and aggregation [31, 32]. Thrombocytopenia, which occurs after cardiac surgery and ECMO implantation, is possibly caused by extensive crosstalk between inflammation, coagulation, bleeding, extracorporeal circuit consumption, and oxidizing stress [8, 33]. Thrombocytopenia has also been proven to be an independent risk factor for poor outcomes in patients undergoing ECMO after cardiac surgery [8, 34]. Persistent, severe thrombocytopenia even indicates a significant physiologic imbalance [34]. Namely, the preserved platelet count of phenotype I also represented a mild inflammatory response and a steady physiology condition, partly reflected by interleukin levels.

As for the treatment process, phenotype I had no significant difference compared with the other clusters in arterial blood lactate level before VA-ECMO implantation. However, with prolonged treatment, the lactate level of phenotype I was the lowest among clusters after both 4 h and 24 h (Appendix Table 2, Fig. 4, Appendix Fig. 9). Lactate behavior is a classic and vital factor related to the in-hospital mortality of critical patients. Several studies have highlighted the independently predicted survival value of CS patients [6, 7, 35, 36]. The lactate scale (<2, 2–8, or >8 mmol/L) has even been identified as an independent predictor of mortality for the REMEMBER score [37]. Respiratory and circulatory function and tissue perfusion of patients in phenotype I recovered promptly, manifested by a gradual decrease in lactate level and partial pressure of oxygen (PO_2_) and a lower level of AST and ALT. This was the direct opposite of the pathophysiological status in phenotype III.

The arterial partial pressure of oxygen trend was similar to the lactate behavior discussed above. Hyperoxia increases oxidative stress, producing free radicals and reactive oxygen species (ROS) and promoting neutrophil activation, which leads to an inappropriate inflammatory response. This effect can be amplified by the complex status of critically ill patients with mechanical circulatory assistance [38]. Recent research confirmed a significant association between hyperoxia and mortality during ECMO [22, 39]. Moreover, Moussa *et al*. [22] found that even a very short exposure to hyperoxia was harmless for patients after receiving ECMO support; our finding also found that patients with a lower level of arterial blood PO_2_ in the first 24 h after VA-ECMO initiation correlated with a favorable prognosis.

Our findings identified two distinct phenotypes among VA-ECMO-treated patients: a hyperinflammatory subtype (Phenotype II) and a renal–hepatic dysfunction subtype (Phenotype III), both associated with poor prognostic outcomes. It is widely approved that inflammatory conditions and oxidative stress could affect the outcome of patients receiving VA-ECMO, as evidenced by the significant production of various inflammatory mediators (such as various interleukins (ILs)) and markers of oxidative stress (such as oxidized low-density lipoprotein (ox-LDL), as well as malondialdehyde (MDA)) [17, 40]. In our analysis, IL-6 and IL-10 were identified through machine learning approaches as key cytokines in profiling the inflammatory status of CS patients undergoing VA-ECMO, revealing distinct cytokine expression patterns across identified phenotypes. Recent evidence, such as the study by Supady *et al*. [40], elucidates that the efficacy of cytokine adsorption in patients with severe COVID-19 pneumonia necessitating ECMO support may not significantly alter survival outcomes. In light of these observations, considering Phenotype II as a potential candidate for cytokine adsorption trials aimed at mitigating inflammatory responses during VA-ECMO treatment must be approached with circumspection. This situation necessitates rigorously designed, targeted clinical trials to unequivocally determine the efficacy of cytokine adsorption in improving patient outcomes. Adopting a methodical approach to evaluating the role of cytokine adsorption in treating CS patients using VA-ECMO highlights the essential need for continued research and evidence gathering.

Phenotype III was characterized by hepatorenal lesions, prone to develop into multiorgan dysfunction and refractory phase with the highest in-hospital mortality. This was consistent with a previous study on cardiogenic shock using the clustering algorithm [14]. “Organ crosstalk” refers to bidirectional interactions between distant organs and summarizes the complex biological communication and feedback between different organs mediated via numerous mechanisms [41]. Renal function and congestion have been identified as important prognostic factors for the outcomes of patients with acute and chronic heart failure [42]. Previous reports found that more than 70% of patients receiving ECMO developed acute kidney injury (AKI), while AKI requiring renal replacement therapy (RRT) in patients undergoing ECMO treatment increased mortality in ICU patients [43, 44]. The liver’s role in oxidant scavenging and antioxidative replenishment may be more susceptible to inflammation and oxidative stress during extracorporeal circulation [10]. In Phenotype III, corrupted hepatic–renal function reflects a refractory tissue perfusion disorder, leading to multiple organ disorder syndrome (MODS) without immediate treatment. Therefore, the timing and standard strategy of RRT or multiple organ support is of great importance for patients with Phenotype III.

There might be a phase overlap between Phenotypes II and III, attributed to the interplay of inflammatory and oxidative stress with organ functionality. Specifically, transitioning from Phenotype II to III could represent an optimal window for VA-ECMO implantation. Despite their distinct clinical presentations, both phenotypes are linked to adverse outcomes. Early recognition of these divergent phenotypes through the discussed variables could offer new insights for clinicians managing CS patients with VA-ECMO, guiding the refinement of intervention strategies. Further verification of these insights necessitates additional cohort studies.

This study’s limitations include its single-center, observational design with a relatively small sample size, constraining the development of a robust validation cohort and limiting the generalizability of the findings. The small dataset also restricts the diversity of variables for cluster analysis, potentially overlooking some characteristics among subtypes. Furthermore, larger, multi-center datasets are necessary for validating the clustering model and exploring additional dimensions of cluster characteristics. Additionally, the predominance of post-cardiotomy patients in our cohort could introduce bias, particularly in the context of inflammatory responses, compared to other VA-ECMO patient groups.

## 5. Conclusions

Utilizing consensus k-means algorithm analysis, this study delineated three distinct phenotypes among VA-ECMO-treated CS patients: “platelet preserved”, “hyperinflammatory”, and “hepatic–renal”. These classifications correlate with specific clinical characteristics and mortality rates, underscoring the importance of early identification. Developing standardized management protocols for these phenotypes could enhance care for patients exhibiting critical conditions.

## Figures and Tables

**Fig. 5.**
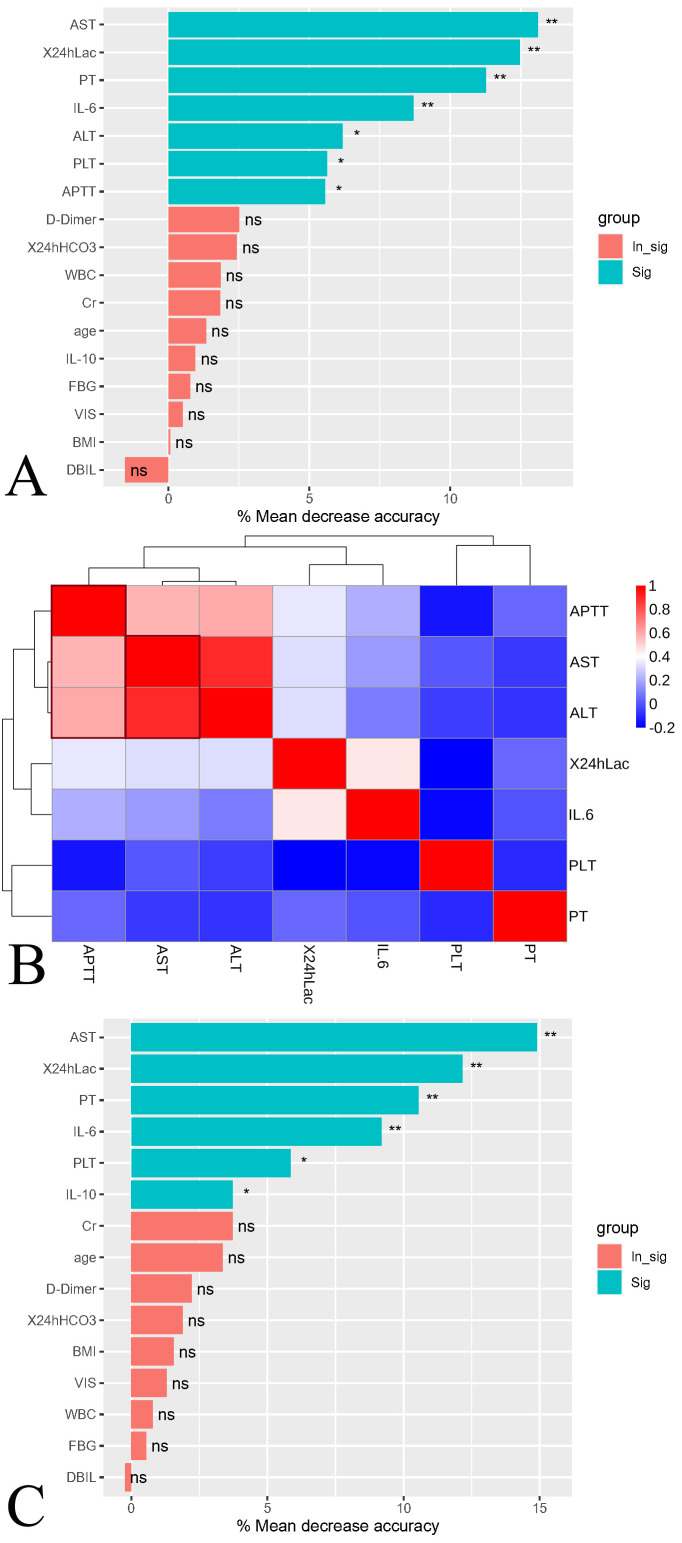
**Selection of cluster-determined variables**. (A) A Random Forest Classifier was trained on in-hospital mortality to identify the most mortality-driving variables. Fig. 2A shows the result using all variables (including collinear variables). (B) Out of the most predictive variables, the correlating (collinear) variables were identified using a correlation matrix, and pairs of correlating (|r| > 0.6) variables with a lower predictive value than the respective other variables (i.e., APTT and ALT) were removed. (C) The five variables with the highest value of predicting in-hospital mortality were the same in both instances (before and after excluding the colinear variables). AST, aspartate aminotransferase; ALT, alanine aminotransferase; PT, prothrombin time; IL-6, Interleukin-6; PLT, platelet count; APTT, activated partial thromboplastin time; WBC, white blood cell count; Cr, creatinine; IL-10, interleukin-10; FBG, fibrinogen; VIS, vasoactive inotropic score; BMI, body mass index; DBIL, direct bilirubin; X24hLac, arterial blood lactate level 24 hours after ECMO initiation; X24hHCO3, arterial blood bicarbonate level 24 hours after ECMO initiation. *, *p* < 0.05; **, *p* < 0.01; ns, no statistically significant difference between the groups.

**Fig. 6.**
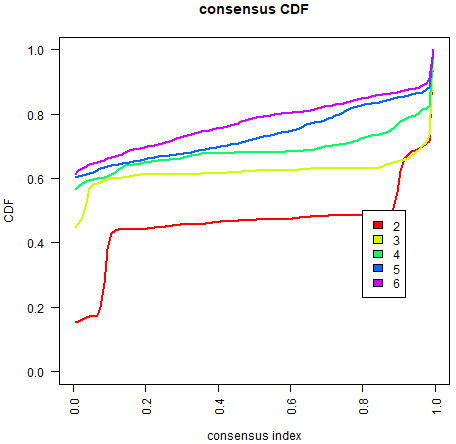
**Cumulative distribution function (CDF) plot for each k to determine where the CDF reaches a maximum without expense of consensus**. Higher a “flatter” curves are favorable (black arrow), and Cluster-Consensus Plot showing the cluster-consensus values of clusters at each k. High values indicate cluster stability, suggesting that 3 may be the optimal choice for the number of clusters.

**Fig. 7.**
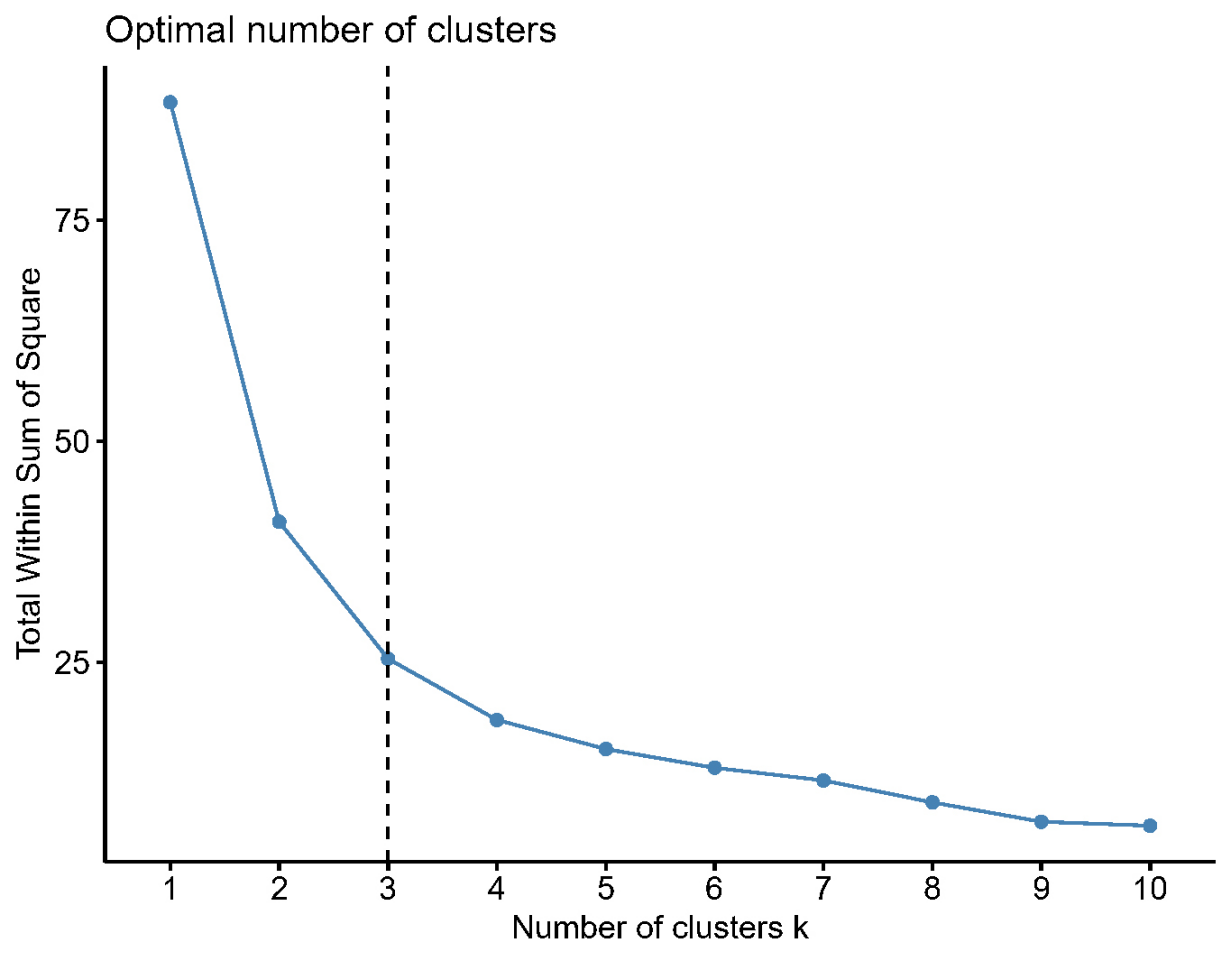
**Elbow method showed optimal k is 3, where the curve was starting to have a diminishing return**.

**Fig. 8.**
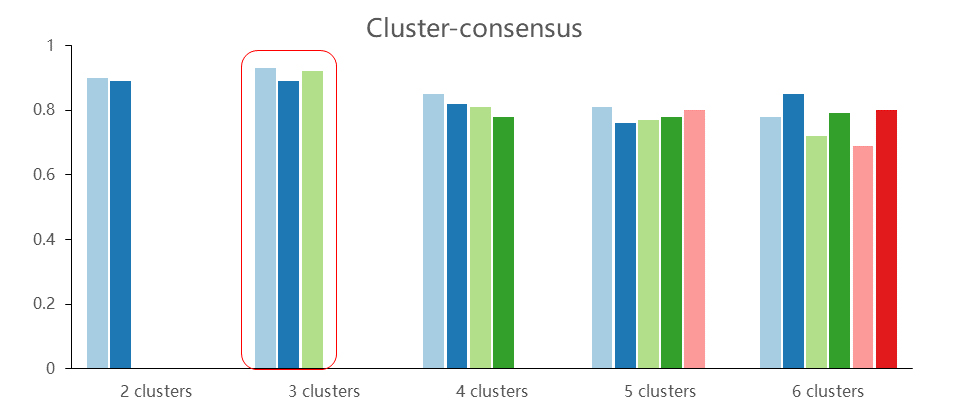
**Cluster-Consensus Plot showing the cluster-consensus values of clusters at each k**. High values indicate cluster stability, suggesting that 3 may be the optimal choice for the number of clusters.

**Fig. 9.**
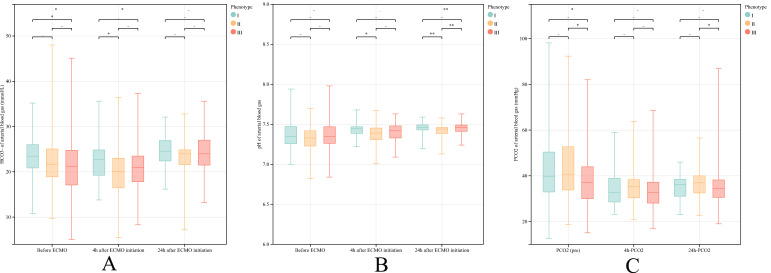
**Phenotype reactions towards ECMO**. A, B and C show the dynamic changes of HCO3- level, pH and PCO_2_ among three phenotypes respectively, indicating separated status towards ECMO support. *p < 0.05, **p < 0.01, ***p < 0.001. ECMO, extracorporeal membrane oxygenation.

**Fig. 10.**
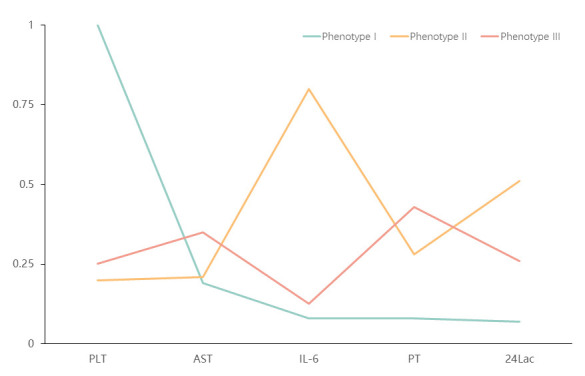
**Tendency of Median Standardized Values of Cluster-determined Variables**. Line charts showed distinct tendency of cluster-determined variables among clusters. PLT, platelet count; AST, aspartic acid transaminase; IL-6, interleukin-6; PT, prothrombin time; 24Lac, arterial blood lactate level 24 hours after ECMO initiation; ECMO, extracorporeal membrane oxygenation.

**Table 2.** **ECMO procedures**.

		All patients	Phenotype I	Phenotype II	Phenotype III	*p* value
			“Platelet preserved”	“Hyper-inflammatory”	“Hepatic-renal”	
		(n = 210)	(n = 36)	(n = 72)	(n = 102)	
Before ECMO						
Echocardiography on admission						
	LVEF, % (IQR)	54.0 (34–64)	49 (27–63)	55 (35–65)	55 (36–64)	0.669
	LVEDD, mm	47.0 (41.0–54.5)	48.5 (44.8–56.8)	45.5 (36.0–53.8)	47.0 (43.0–53.0)	0.184
NYHA on admission, n (%)						0.508
	I–II	54 (25.7)	11 (30.6)	21 (29.2)	22 (21.6)	
	III	130 (61.9)	19 (52.8)	42 (58.3)	69 (67.6)	
	IV	26 (12.4)	6 (16.7)	9 (12.5)	11 (10.8)	
Cardiac arrest before ECMO, n (%)		37 (17.6)	4 (11.1)	13 (18.1)	20 (19.6)	0.498
SOFA score, median (IQR)		10 (8–10)	9 (7–10) ^𝑎^	10 (9–11) ^𝑏^	10 (9–11) ^b^	0.001
VIS, median (IQR)		22.0 (11.8–40.0)	24.0 (10.0–32.4)	20.0 (12.0–45.0)	23.0 (11.8–36.5)	0.977
ABG, median (IQR)						
	pH	7.35 (7.25–7.45)	7.35 (7.25–7.48)	7.33 (7.22–7.42)	7.35 (7.26–7.47)	0.246
	HCO_3_^–^, mmol/L	21.7 (18.4–25.8)	23.5 (20.8–26.0) ^𝑎^	21.8 (18.9–26.0) ^𝑎𝑏^	21.3 (17.1–24.9) ^𝑏^	0.030
	PO_2_, mmHg	80.0 (51.0–164.6)	54.4 (46.2–125.6)	70.7 (51.6–185.2)	94.4 (54.1–176.9)	0.211
	PCO_2_, mmHg	38.9 (32.0–17.9)	39.8 (32.5–50.5) ^a⁢b^	40.6 (33.7–52.9) ^𝑎^	37.1 (30.0–44.0) ^𝑏^	0.044
	Lactate, mmol/L	9.1 (4.7–13.7)	6.7 (3.3–9.6) ^𝑎^	7.1 (3.5–12.8) ^𝑎^	9.9 (6.0–16.1) ^𝑏^	0.001
After ECMO						
ECMO flow, L/min, mean ± SD						
	4 h	3.4 ± 0.6	3.3 ± 0.6	3.4 ± 0.6	3.4 ± 0.5	0.638
	12 h	3.5 ± 0.5	3.3 ± 0.6	3.5 ± 0.5	3.4 ± 0.5	0.058
	24 h	3.1 ± 0.3	3.4 ± 0.5	3.4 ± 0.6	3.4 ± 0.5	0.589
24 h Vital signs, median (IQR)						
	Temperature, °C	36.6 (36.3–36.9)	36.6 (36.5–36.9)	36.6 (36.3–37.0)	36.6 (36.2–36.9)	0.387
	Heart rate, beats/min	95 (83–101)	95 (91–100)	95 (84–102)	92 (80–101)	0.118
	Respiratory rate, breaths/min	12 (10–13)	12 (12–13)	12 (10–13)	12 (10–13)	0.360
	MAP, mmHg	71 (66–80)	71 (70–82)	71 (59–80)	71 (65–80)	0.367
4 h ABG, median (IQR)						
	pH	7.40 ± 0.11	7.43 ± 0.09	7.38 ± 0.12	7.39 ± 0.12	0.126
	HCO_3_^–^, mmol/L	20.9 (17.6–23.9)	22.8 (19.0–25.0) ^𝑎^	20.1 (16.4–23.1) ^𝑏^	21.0 (17.8–23.5) ^𝑎𝑏^	0.045
	PO_2_, mmHg	150.5 (93.7–266.6)	98.4 (76.0–137.5) ^𝑎^	170.7 (113.7–286.2) ^𝑏^	165.1 (92.0–289.3) ^𝑏^	0.001
	PCO_2_, mmHg	33.4 (28.5–37.9)	32.8 (28.5–39.0)	35.3 (30.3–38.3)	32.8 (28.0–37.3)	0.303
	Lactate, mmol/L	9.4 (5.0–14.9)	5.4 (2.6–8.5) ^𝑎^	11.3 (6.5–14.8) ^𝑏^	10.3 (5.6–17.0) ^𝑏^	<0.001
24 h ABG, median (IQR)						
	pH	7.45 (7.41–7.48)	7.46 (7.43–7.50) ^𝑎^	7.44 (7.38–7.46) ^𝑏^	7.46 (7.41–7.49) ^𝑎^	0.007
	HCO_3_^–^, mmol/L	24.0 (21.6–26.6)	24.6 (22.4–27.0)	24.0 (21.6–25.0)	24.0 (21.5–27.0)	0.367
	PO_2_, mmHg	125.2 (83.1–181.8)	100.2 (75.3–126.8) ^𝑎^	129.6 (82.7–160.1) ^𝑏^	141.6 (96.4–215.5) ^𝑏^	0.003
	PCO_2_, mmHg	35.5 (30.8–39.0)	36.2 (31.0–38.5)	37.0 (31.8–40.0)	34.4 (30.4–38.4)	0.128
	Lactate, mmol/L	3.1 (2.1–4.8)	2.2 (1.4–3.0) ^𝑎^	4.3 (2.1–5.2) ^𝑏^	3.4 (2.2–5.1) ^𝑏^	<0.001
Platelet transfusion, mL/kg/d		1 (0–2)	1 (0–2)	1 (0–2)	1 (0–2)	0.424
FFP transfusion, mL/kg/d		2 (1–4)	2 (1–3)	2 (1–4)	2 (1–5)	0.231
RBC transfusion, mL/kg/d						

a, b: Based on the result of Bonfferroni method after chi-square test or the result of LSD method after nonparametric test, same letters in the horn markers manifested no significance between phenotypes, while different letters in the horn markers indicated statistically significant. IQR, interquartile range; LVEF, left ventricular ejection fraction; LVEDD, left ventricular end-diastolic diameter; NYHA, New York Heart Association Functional Classification; SOFA, Sequential Organ Failure Assessment; VIS, vasoactive inotropic score; ABG, arterial blood gas; ECMO, extracorporeal membrane oxygenation; MAP, mean arterial pressure; FFP, fresh frozen plasma; RBC, red blood cell; LSD, Least Significant Difference.

**Table 3.** **Laboratory tests of 24 hours after ECMO initiation**.

		All patients	Phenotype I	Phenotype II	Phenotype III	*p* value
			“Platelet preserved”	“Hyper-nflammatory”	“Hepatic-enal”	
		(n = 210)	(n = 36)	(n = 72)	(n = 102)	
Complete blood count						
	White blood cell count, ×10^9^/L	11.75 (8.13–17.18)	11.98 (9.43–15.85) ^a⁢b^	10.50 (6.35–14.55) ^a^	13.16 (8.44–19.20) ^𝑏^	0.007
	Platelets, ×10^9^/L	58 (36–96)	106 (73–165) ^𝑎^	51 (35–85) ^𝑏^	55 (32–86) ^b^	<0.001
	Neutrophil count, ×10^9^/L	11.80 (7.72–13.12)	11.80 (9.22–13.07) ^𝑎𝑏^	10.36 (6.05–11.80) ^a^	11.80 (7.90–16.76) ^𝑏^	0.009
	Lymphocyte count, ×10^9^/L	0.95 (0.58–1.03)	0.92 (0.57–0.95)	0.95 (0.65–1.20)	0.85 (0.55–1.00)	0.094
	Monocyte count, ×10^9^/L	0.55 (0.32–0.61)	0.55 (0.52–0.67) ^𝑎^	0.50 (0.22–0.55) ^𝑏^	0.54 (0.32–0.63) ^a⁢b^	0.031
	Red blood cell count, ×10^12^/L	2.78 (2.43–2.95)	2.78 (2.68–3.12) ^𝑎^	2.71 (2.27–2.79) ^𝑏^	2.70 (2.48–3.10) ^a⁢b^	0.021
	Hemoglobin, g/dL	84 (72–89)	84 (81–93) ^𝑎^	83 (70–85) ^𝑏^	84 (75–92) ^𝑎𝑏^	0.024
	Hematocrit, %	24.6 (21.5–26.0)	24.6 (23.7–26.5) ^𝑎^	24.3 (20.4–24.6) ^𝑏^	24.6 (21.5–26.6) ^a⁢b^	0.026
	MCV, fL	87.8 (86.3–89.4)	87.8 (86.3–89.3)	87.8 (86.8–90.0)	87.8 (86.1–89.4)	0.865
	MCH, pg	30.6 (29.9–31.1)	30.6 (29.7–30.7)	30.6 (30.1–31.1)	30.6 (29.7–31.4)	0.345
	MCHC, g/L	343 (340–353)	343 (339–353)	343 (342–352)	343 (340–354)	0.708
	RDW–CV, %	14.3 (13.5–14.7)	14.3 (13.4–14.3)	14.3 (13.6–14.9)	14.2 (13.4–14.6)	0.225
Hepatic–renal function						
	AST, U/L	210 (95–599)	85 (48–142) ^𝑎^	150 (61–307) ^𝑏^	494 (220–2234) ^𝑐^	<0.001
	ALT, U/L	78 (25–341)	23 (18–59) ^𝑎^	42 (19–132) ^𝑏^	223 (71–1301) ^c^	<0.001
	TBIL, µmol/L	34.4 (18.8–38.9)	34.3 (21.1–38.9)	28.0 (15.8–39.0)	39.0 (20.0–56.1)	0.065
	DBIL, µmol/L	13.0 (7.9–29.6)	9.2 (5.9–20.4) ^𝑎𝑏^	12.9 (7.3–23.8) ^𝑎^	14.3 (9.0–42.5) ^𝑏^	0.029
	Creatinine, µmoI/L	121.4 (86.3–158.7)	86.5 (67.4–127.7) ^𝑎^	121.0 (87.8–157.9) ^𝑏^	137.3 (93.6–188.8) ^𝑏^	<0.001
Coagulation						
	PT, second	16.7 (13.8–21.4)	14.6 (13.2–17.8) ^𝑎^	16.5 (14.2–20.6) ^𝑎𝑏^	18.3 (14.5–24.6) ^𝑏^	0.002
	APTT, second	48.5 (35.8–65.5)	41.6 (32.6–56.3) ^𝑎^	56.4 (39.9–81.4) ^𝑏^	45.0 (35.3–63.0) ^𝑎^	0.004
	D–Dimer,	1008 (449–3000)	738 (390–2043) ^a^	755 (435–2333) ^a^	1309 (502–4717) ^𝑎^	0.047
	Fibrinogen, g/L	2.2 (1.4–3.2)	2.7 (2.2–3.6) ^𝑎^	2.0 (1.3–3.2) ^𝑎𝑏^	1.9 (1.4–3.1) ^𝑏^	0.018
Inflammation						
	IL–6, Pg/ml	93.4 (26.1–601.0)	17.5 (4.7–44.7) ^a^	799.6 (266.0–1757.3) ^b^	66.1 (23.6–148.3) ^𝑐^	<0.001
	IL–10, Pg/mL	14.1 (4.6–58.8)	4.0 (1.5–7.0) ^𝑎^	29.5 (9.2–82.5) ^𝑏^	18.2 (5.4–73.7) ^𝑏^	<0.001

a, b, c: Based on the result of Bonfferroni method after chi-square test or the result of LSD method after nonparametric test, same letters in the horn markers manifested no significance between phenotypes, while different letters in the horn markers indicated statistically significant. MCV, mean corpuscular volume; MCH, mean corpuscular hemoglobin; MCHC, mean corpuscular hemoglobin concentration; RDW-CV, coefficient of variation of red blood cell distribution width; TBIL, total bilirubin; DBIL, direct bilirubin; PT, prothrombin time; APTT, activated partial thromboplastin time; AST, aspartate aminotransferase; ALT, alanine aminotransferase; IL, interleukin; Cr, creatinine; LSD, Least Significant Difference; ECMO, extracorporeal membrane oxygenation.

**Table 4.** **Outcomes**.

		All patients	Phenotype I	Phenotype II	Phenotype III	*p* value
			“Platelet preserved”	“Hyper-nflammatory”	“Hepatic-enal”	
		(n = 210)	(n = 36)	(n = 72)	(n = 102)	
In-hospital mortality		104 (49.5)	9 (25.0) ^a^	38 (52.8) ^b^	57 (55.9) ^b^	0.005
Successful weaning from ECMO		142 (67.6)	30 (83.3)	45 (62.5)	67 (65.7)	0.078
ECMO duration (hour)		105.4 (66.7–153.6)	98.5 (87.4–132.0)	105.0 (48.9–147.9)	108.0 (68–183)	0.310
MV duration (hour)		190.7 (82.4–269.9)	193.5 (99.9–302.1)	105.4 (66.6–153.6)	150.7 (90.5–285.2)	0.547
Complications						
	Bleeding at intubation site	25 (13)	2 (6.1)	8 (11.9)	15 (16.1)	0.319
	Limb ischemia required fasciotomy	9 (4.3)	1 (2.8)	4 (5.6)	4 (1.9)	0.773
	Cr >3.0 mg/dL	32 (15.2)	1 (2.8)	11 (15.3)	20 (19.6)	0.054
	Hyperbilirubinemia	104 (49.5)	5 (13.9) ^a^	43 (59.7) ^b^	56 (54.9) ^b^	<0.001
	Neurological complications	15 (7.1)	3 (8.3)	7 (9.7)	5 (4.9)	0.456
	Hospital length of stay (day)	20 (12–28)	20 (15–30)	16 (10–26)	21 (13–29)	0.096
	ICU length of stay (day)	9 (5–14)	10 (6–14)	9 (3–13)	10 (4–15)	0.231

a, b, c: Based on the result of Bonfferroni method after chi-square test or the result of LSD method after nonparametric test, same letters in the horn markers manifested no significance between phenotypes, while different letters in the horn markers indicated statistically significant. Neurological complications including cerebral hemorrhage, cerebral infarction, epileptic seizure, and cerebral death. Hyperbilirubinemia was defined as DBIL >2 mg/dL, IBIL >13 mg/dL, or TBIL >15 mg/dL. ICU, intensive care unit; ECMO, extracorporeal membrane oxygenation; DBIL, direct bilirubin; TBIL, total bilirubin; LSD, Least Significant Difference; MV, mechanical ventilation; IBIL, indirect bilirubin; Cr, creatinine.

**Table 5.** **Details of patients receiving cardiac surgery**.

		All patients	Phenotype I	Phenotype II	Phenotype III	*p* value
			“Platelet preserved”	“Hyper-nflammatory”	“Hepatic-enal”	
		(n = 210)	(n = 36)	(n = 72)	(n = 102)	
Surgery in this hospitalization, n (%)		184 (87.6)	27 (75.0) ^𝑎^	70 (97.2) ^𝑏^	87 (85.3) ^𝑎^	0.003
Surgery under CPB, n (%)		144 (68.6)	22 (61.1)	57 (79.2)	65 (63.7)	0.055
Type of surgery, n (%)						
	CABG	71 (33.8)	8 (22.2) ^a^	32 (44.4) ^𝑏^	31 (30.4) ^b^	0.040
	Valve procedure	61 (29.0)	11 (30.6)	23 (31.9)	27 (26.5)	0.708
	CABG + valve procedure	26 (12.4)	3 (8.3)	9 (12.5)	14 (13.7)	0.700
	Repair of acute aortic dissection	12 (5.7)	1 (2.8)	5 (6.9)	6 (5.9)	0.732
	Repair of acute aortic dissection + CABG	7 (3.3)	0 (0)	2 (2.8)	5 (4.9)	0.534
	Pulmonary embolectomy	6 (2.9)	1 (2.8)	2 (2.8)	3 (2.9)	1.000
	Heart transplantation	9 (4.3)	3 (8.3)	1 (1.4)	5 (4.9)	0.169
	Others	11 (5.2)	1 (2.8)	5 (6.9)	5 (4.9)	0.770

a, b: Based on the result of the Bonferroni method after chi-square test or the result of LSD method after nonparametric test, the same letters in the horn markers indicate no significance between clusters, while different letters in the horn markers indicate statistically significant. CPB, cardiopulmonary bypass; CABG, coronary artery bypass graft; LSD, Least Significant Difference.

## Data Availability

The clinical data collected in this study have been uploaded to the database of the Extracorporeal Life Support Professional Committee of Chinese Medical Doctor Association, while biospecimen data are stored at the Biomedical Innovation Center of Beijing Shijitan Hospital, affiliated with Capital Medical University. Access to these datasets requires a formal application, and interested readers may contact the corresponding author to obtain permission for data access based on their specific research needs.

## Abbreviations

VA-ECMO, venous–arterial extracorporeal membrane oxygenation; CS, cardiogenic shock; PLT, platelet count; AST, aspartic acid transaminase; IL-6, interleukin-6; PT, prothrombin time; ML, machine learning; SAVE, survival after VA-ECMO score; AMI, acute myocardial infarction; REMEMBER, pRedicting mortality in patients undergoing veno–arterial Extracorporeal MEMBrane oxygenation after coronary artEry bypass gRafting; ENCOURAGE, prEdictioN of Cardiogenic shock OUtcome foR Acute myocardial infarction patients salvaGed by VA-ECMO score; ARDS, acute respiratory distress syndrome; LVEF, left ventricular ejection fraction; LVEDD, left ventricular end-diastolic diameter; NYHA, New York Heart Association Functional Classification; VIS, the vasoactive inotropic score; ABG, arterial blood gas; MAP, mean arterial pressure; TSNE plot, t-distributed stochastic neighbor embedding plot; IQR, interquartile range; CRRT, continuous renal replacement therapy; SOFA, The Sepsis-related Organ Failure Assessment score; ROS, reactive oxygen species; ox-LDL, oxidized low-density lipoprotein; MDA, malondialdehyde; MODS, multiple organ disorder syndrome; IABP, intra-aortic balloon pump; CPB, cardiopulmonary bypass; CABG, coronary artery bypass grafting; ICU, intensive care unit.

## Appendix 1. Details of Part of Method

### Details of ECMO Management

VA-ECMO support was initiated via peripheral cannulation via the femoral route, using semi-open or percutaneous methods. An additional 6 Fr catheter was inserted distally into the femoral artery to prevent severe leg ischemia. Clinical assessments were used to adjust ECMO blood flow (e.g., mixed venous oxygen saturation, evidence of hypoperfusion, resolution of hyperlactatemia, and normalization of mean arterial pressure). Unfractionated heparin was administered intravenously to maintain an activated clotting time of 180–210 s or an activated partial thromboplastin time of 1.5–2 times normal. The complications associated with ECMO were closely monitored. Patients who met our published institutional weaning criteria and passed an ECMO weaning trial consisting of decreasing and clamping the ECMO flow were given ECMO weaning [45, 46].

### Supplements of Selecting Cluster-Determined Variables

#### VIS

VIS was calculated when the vasoactive agents adequately maintained a relatively stable hemodynamic status according to the following formula: dosages of dopamine (in µg/kg^-1^/min^-1^) + dosages of dobutamine (in µg/kg^-1^/min^-1^) + [dosages of epinephrine (in µg/kg^-1^/min^-1^) + norepinephrine (in µg/kg^-1^/min^-1^)] × 100 + dosages of pituitrin(in unit/min) * 100 + dosages of milrinone (in µg/kg^-1^/min^-1^) * 15 [17].

### Details of Algorithm of Selecting Cluster-Determined Variables

A previous study recommended that the minimal sample size of cluster analysis was more than 2^n^ cases (n = number of variables), and 5 × 2^n^ would be favorable [47], So a random forest classifier was used to identify important variables according to mortality association before applying the clustering algorithm. Because the random forest classifier could not identify colinear variables, we first ran the random forest classifier with all variables of interest to ascertain their predictive value for in-hospital mortality, and then trained the random forest classifier again after the removal of correlating variables and identified the most predictive variables for the subsequent clustering process.

### Details of Ascertaining the Number of Clusters (k)

Selecting optimal cluster-determined covariates was the first step of cluster analysis. Before starting the consensus k-means analysis, the number of clusters (k) was confirmed using the random forest classifier and several other methods (cumulative distribution function plot, cluster-consensus plot, elbow plot, and TSNE plot). In the present study, a k-value of 3 was considered favorable.

## Appendix 2. Details of part of Result

### Detailed procedures of Clusters identification

Seven significant variables were obtained (AST, 24-hour lactate level, PT, IL-6, ALT, platelet count, and APTT) (Appendix Fig. 5A). As the linear relationships between the seven variables might affect the reliability of the consensus k-means algorithm analysis, a correlation test was performed to eliminate the non-orthogonal variables and reduce the dimensionality of the model. Two variables (APTT and ALT) were excluded (Appendix Fig. 5B). The five highest predictive-value variables were then included in further cluster analysis (Appendix Fig. 5C).

## Appendix 3

See Tables 2,3,4,5, Figs. 5,6,7,8,9,10.
